# Multiscale, patient-specific computational fluid dynamics models predict formation of neointimal hyperplasia in saphenous vein grafts

**DOI:** 10.1016/j.jvscit.2019.09.009

**Published:** 2020-04-12

**Authors:** Francesca Donadoni, Cesar Pichardo-Almarza, Shervanthi Homer-Vanniasinkam, Alan Dardik, Vanessa Díaz-Zuccarini

**Affiliations:** a Department of Mechanical Engineering, Multiscale Cardiovascular Engineering Group, University College London, London, United Kingdom; b Certara Quantitative Systems Pharmacology (QSP), Canterbury, United Kingdom; c Wellcome/EPSRC Centre for Interventional and Surgical Sciences, University College London, London, United Kingdom; d Yale University School of Medicine, Vascular Biology and Therapeutics, New Haven, Conn; e Department of Surgery, VA Connecticut Healthcare Systems, West Haven, Conn

**Keywords:** Neointimal hyperplasia, Vein grafts, Shear stress, Computational fluid dynamics, Multiscale modeling

## Abstract

Stenosis due to neointimal hyperplasia (NIH) is among the major causes of peripheral graft failure. Its link to abnormal hemodynamics in the graft is complex, and isolated use of hemodynamic markers is insufficient to fully capture its progression. Here, a computational model of NIH growth is presented, establishing a link between computational fluid dynamics simulations of flow in the lumen and a biochemical model representing NIH growth mechanisms inside the vessel wall. For all three patients analyzed, NIH at proximal and distal anastomoses was simulated by the model, with values of stenosis comparable to the computed tomography scans.

Autogenous vein bypass is the most common technique for peripheral artery revascularization for severe peripheral artery diseases but is prone to development of neointimal hyperplasia (NIH), a leading cause of bypass failure.^1^ Both experimental studies and clinical observations suggest that one of the factors destabilizing the remodeling process is a lower level of wall shear stress on the arterial wall,^2^ and numerous computational fluid dynamics (CFD) studies of blood flow have used shear stress indices—including time-averaged wall shear stress (TAWSS) and oscillatory shear index (OSI), for example—as markers to identify potentially problematic areas of vascular remodeling (Supplementary Table I).^3^^,^^4^

We hypothesized that both low and oscillatory levels of shear stress should be considered simultaneously in assessing the proclivity of a certain region in bypass grafts to develop NIH,^5^^,^^6^ and we simulated NIH progression using a multiscale computational framework that we previously developed,^7^ comparing our results with a patient-specific clinical data set (obtained with the patients' informed consent for research and publication).

## Methods

The computational framework was informed by patient-specific imaging data, including anatomic images and hemodynamic markers. Duplex ultrasound scans immediately after surgery and computed tomography (CT) scans at 8, 19, and 24 months after surgery (for patients 1-3, respectively) were obtained from routine clinical studies between August and November 2015 after approval from the Institutional Review Board (AD0009, Veterans Affairs Connecticut Healthcare System, West Haven, Conn), and all data required deidentification despite Institutional Review Board approval in compliance with Veterans Affairs requirements for patients' privacy.

Duplex ultrasound images and CT scans were patient specific (not standardized). CT scans immediately after surgery were not available as the data were acquired retrospectively from selected patients who received standard of care with regular postoperative surveillance. Postoperative surveillance, at the institution where data were collected, is performed only by duplex ultrasound as it is noninvasive, does not require nephrotoxic dye, is reproducible, and correlates with outcomes as documented with extensive literature, and as such, CT scans are obtained only when the duplex ultrasound examination suggests an abnormality and additional anatomic information is required. In our analysis, a full three-dimensional image of the artery graft geometry was needed to compare the results of simulations with the patient-specific cases, and duplex ultrasound images were not sufficient for this purpose. To overcome these limitations, a “baseline” configuration (representing the vein graft conditions right after implantation) was obtained by processing the images and “virtually removing” regions of NIH growth, well in line with other work in the literature.^8^, ^9^, ^10^, ^11^, ^12^, ^13^, ^14^, ^15^

**Supplementary Fig 1:**
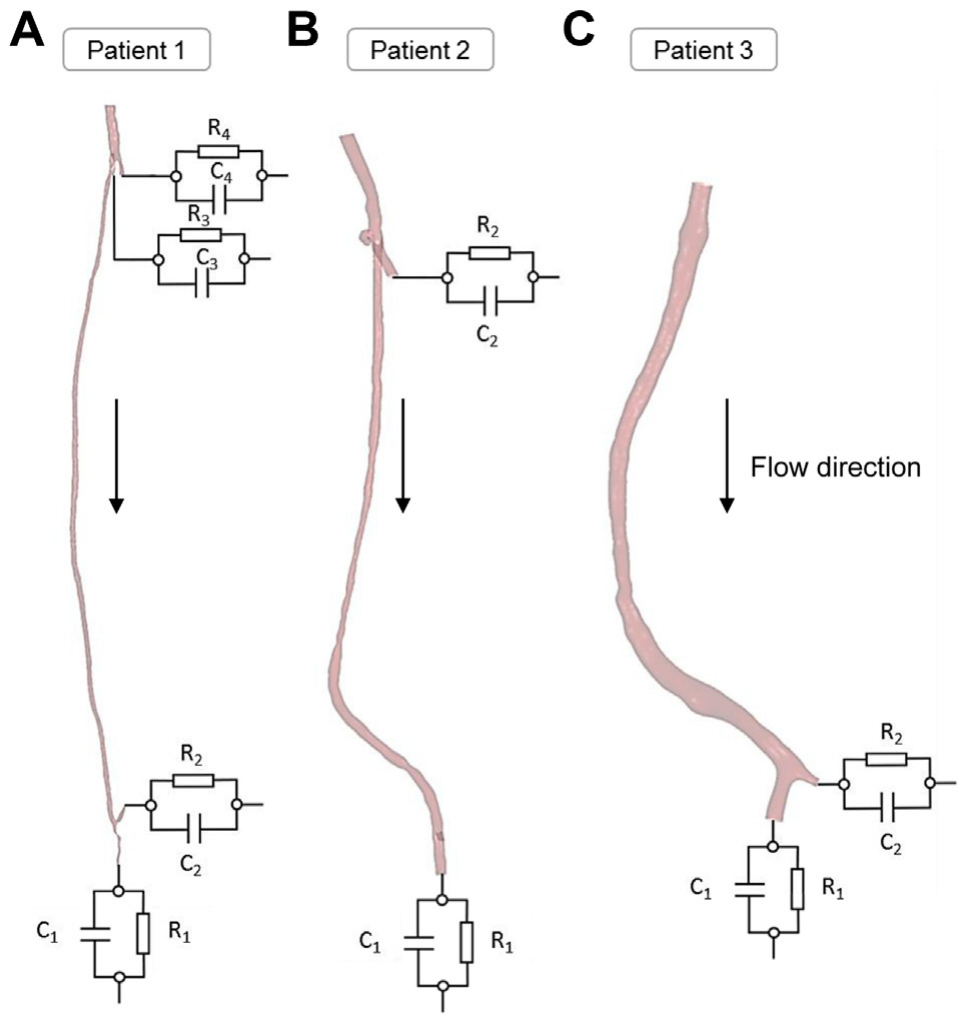
Boundary conditions at the outlets were modeled using RC elements. **A,** Patient 1, femoral-distal bypass. **B,** Patient 2, femoral-popliteal bypass. **C,** Patient 3, femoral-popliteal bypass.

CFD analyses were performed as described in a previous publication.^7^ A non-newtonian Carreau-Yasuda model was used for blood viscosity, with parameters reported in previous studies.^16^ For comparison, simulations were also run with a Newtonian model (viscosity of 0.035 dyn∙s/cm^2^). Inflow conditions were obtained from duplex ultrasound scans through image processing, converting duplex ultrasound images of velocity at the inlet locations into mass flow rate curves using MATLAB (MathWorks, Natick, Mass). These were applied first with a flat profile and with a parabolic profile in a different set of simulations for comparison. Boundary conditions at the outlets were implemented through two-element Windkessel models of the external vasculature (Supplementary Fig 1), tuned to patient-specific data on a zero-dimensional model (Supplementary Table II).

**Supplementary Fig 2:**
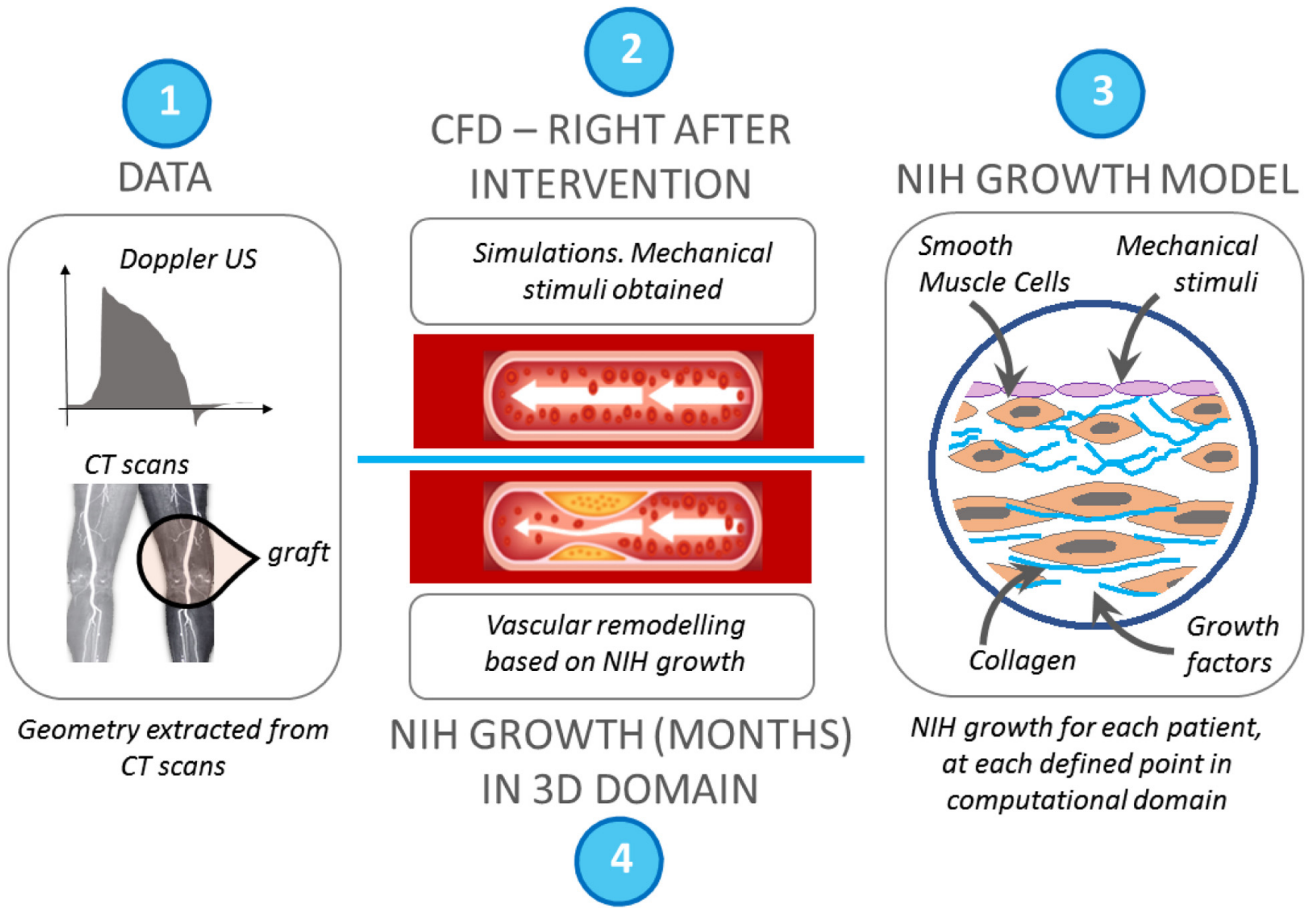
A schematic diagram of the framework for simulating neointimal hyperplasia (*NIH*) progression. *1*, Clinical data are extracted for each patient (eg, computed tomography [*CT*] scans, Doppler ultrasound [*US*] images) and processed for computational fluid dynamics (*CFD*) simulations. *2*, CFD simulations are performed right after the intervention, obtaining mechanical stimuli and calculating hemodynamic stress indices (eg, time-averaged wall shear stress [TAWSS], oscillatory shear index [OSI], highly oscillatory, low magnitude shear [HOLMES]). *3*, Simulations of a biochemical model of NIH growth and progression. *4*, Simulations of NIH growth for a long time span in the three-dimensional (*3D*) domain.

Simulation results were processed using ANSYS CFD-Post (Ansys, Canonsburg, Pa). Hemodynamic stress indices linked to vascular remodeling, specifically TAWSS and a term encompassing low shear together with oscillations, the highly oscillatory, low magnitude shear (HOLMES; Supplementary Table I, were extracted at each node and imported into a mathematical model of NIH progression, described in Supplementary Fig 2. The output of the simulation model for each patient is the predicted (calculated) value of NIH growth along the graft, following the same blueprint described in our previous work^7^ and summarized in Supplementary Table III. Based on the “base” configuration for each patient (a vein graft free of NIH, representing conditions just after implantation), CFD analyses are coupled to a mathematical (time-dependent) model of NIH growth. This is a dynamic simulation process that captures the transformation of the vein graft due to NIH and mimics the evolution of the disease for each patient in time. The model is a mechanistic system of equations that does not impose constant intimal growth but uses mathematical representations of basic biologic mechanisms to simulate NIH development on each patient-specific case. The model is based on several assumptions. One assumption is that while the volume of *intima* grows, the *media* remains constant. In addition, disease progression is assumed to be stimulated by four main mechanisms (which are modeled through equations linked to wall shear stress): smooth muscle cell and collagen turnover, growth factors, and nitric oxide production. Each mechanism is described through the use of ordinary differential equations with parameters obtained from experimental data previously published, as described in previous work.^7^

Following the biochemical model simulations, a geometry with NIH growth is obtained. We measured cross-sectional areas of the lumen where stenosis due to NIH was most severe in all the cases analyzed by subtracting the final result from the baseline configuration and compared each case with CT scans.

**Supplementary Fig 3:**
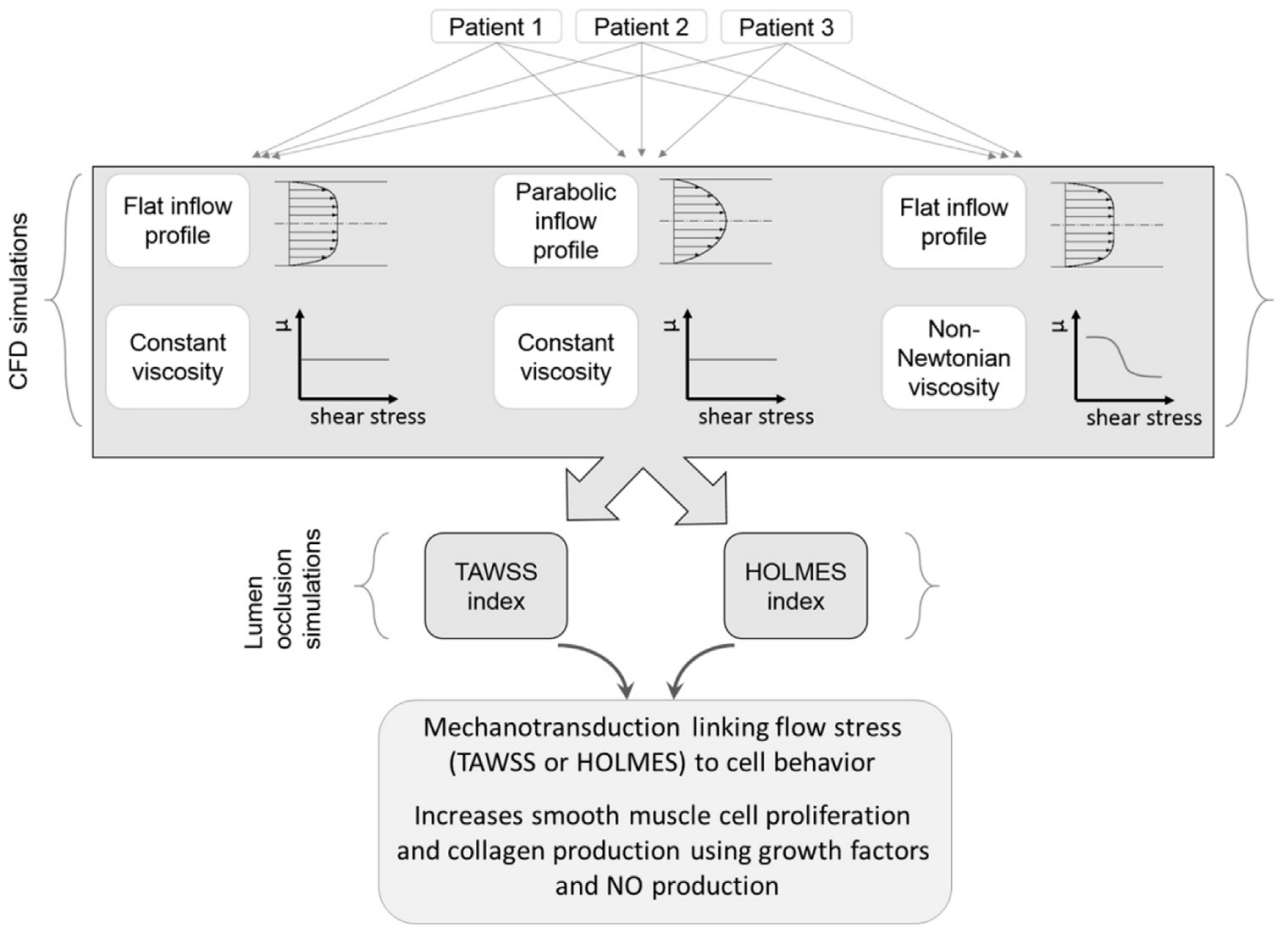
Overview of interactions. A complex interaction between mechanical stimuli on the wall affects cell behavior, with smooth cells and collagen proliferating because of growth factors and nitric oxide (*NO*) production. The result is the development of neointimal hyperplasia (NIH) and vascular remodeling. Details of the model are published in reference 14. *CFD,* Computational fluid dynamics; *HOLMES,* highly oscillatory, low magnitude shear; *TAWSS,* time-averaged wall shear stress.

A diagram of the different cases presented in this analysis is shown in Supplementary Fig 3.

## Results

**Fig 1:**
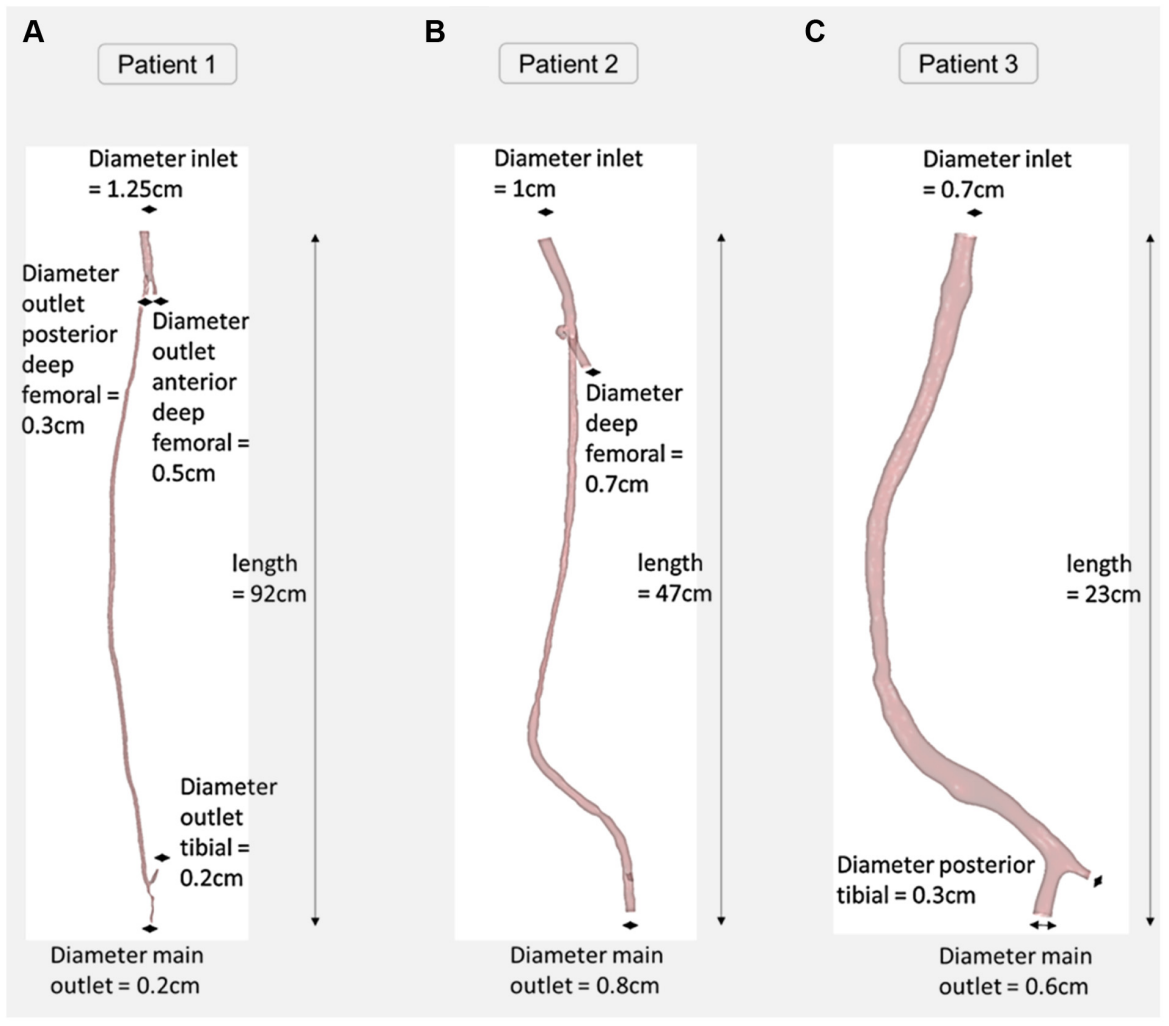
Summary of the geometric characteristics of patients analyzed in the study. **A,** Patient 1, femoral-distal bypass. **B,** Patient 2, femoral-popliteal bypass. **C,** Patient 3, femoral-popliteal bypass.

**Fig 2:**
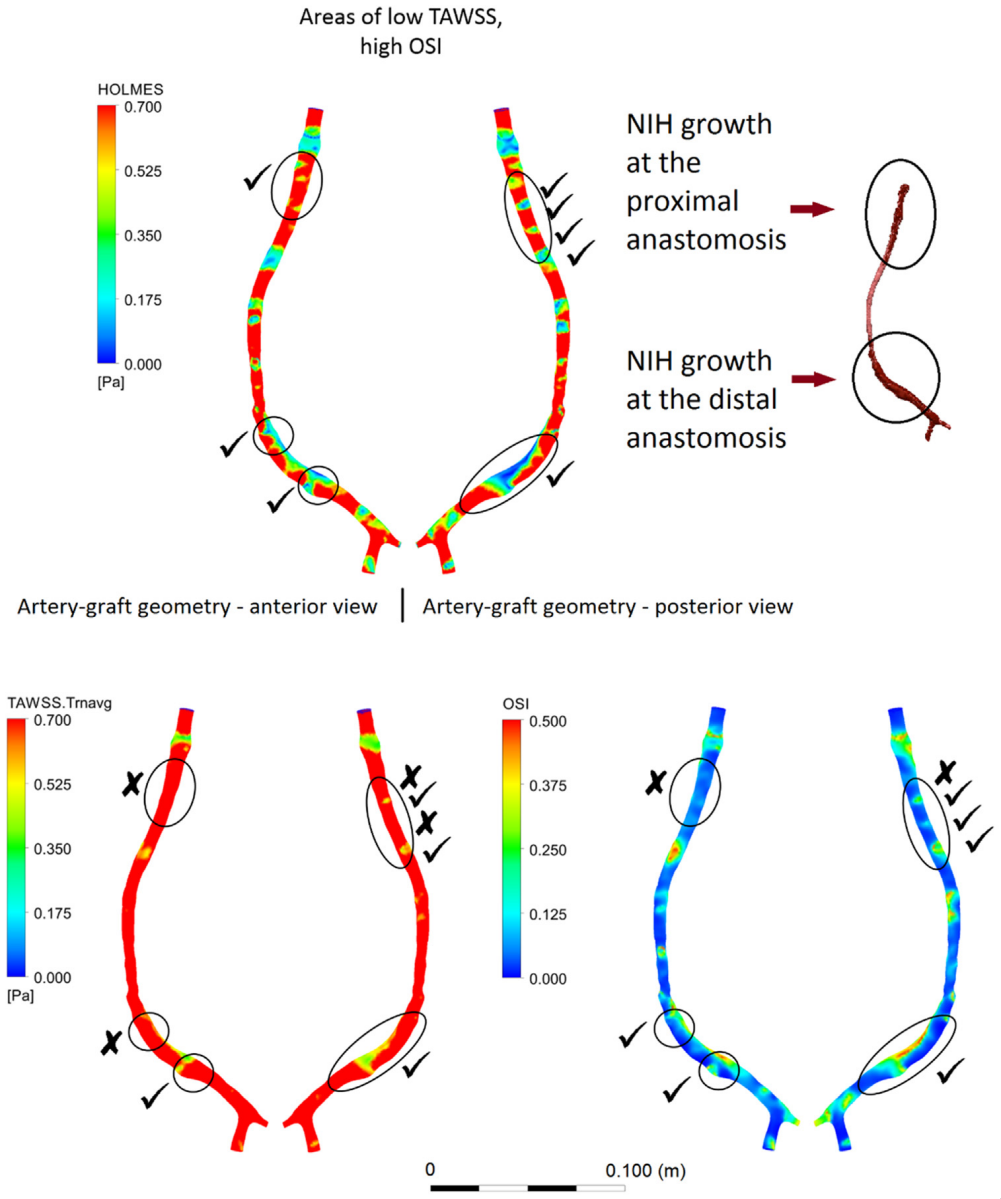
Example of contour plots of hemodynamic indices obtained for patient 3. **A,** Simulation for highly oscillatory, low magnitude shear (*HOLMES*). **B,** Simulation for time-averaged wall shear stress (*TAWSS*). **C,** Simulation for oscillatory shear index (*OSI*). The *tick marks* refer to areas of neointimal hyperplasia (*NIH*) actually captured by computational fluid dynamics (CFD) simulations. TAWSS and HOLMES are considered to have low values when <0.5 Pa (the *non-red* areas in the contour plots). It can be seen that in the case of TAWSS and OSI, many remodeled regions are not indicated (identified by *x*).

Hemodynamic analyses were performed on all three bypass geometries (Fig 1). As shown in Fig 2, at the proximal and distal anastomosis where NIH developed, low values of TAWSS (<0.5 Pa) did not always correspond to regions of NIH progression. Similarly, high values of OSI did not always fully capture the areas of NIH progression either. HOLMES was the only index able to capture all regions of NIH progression (Fig 2, *A*).

**Fig 3:**
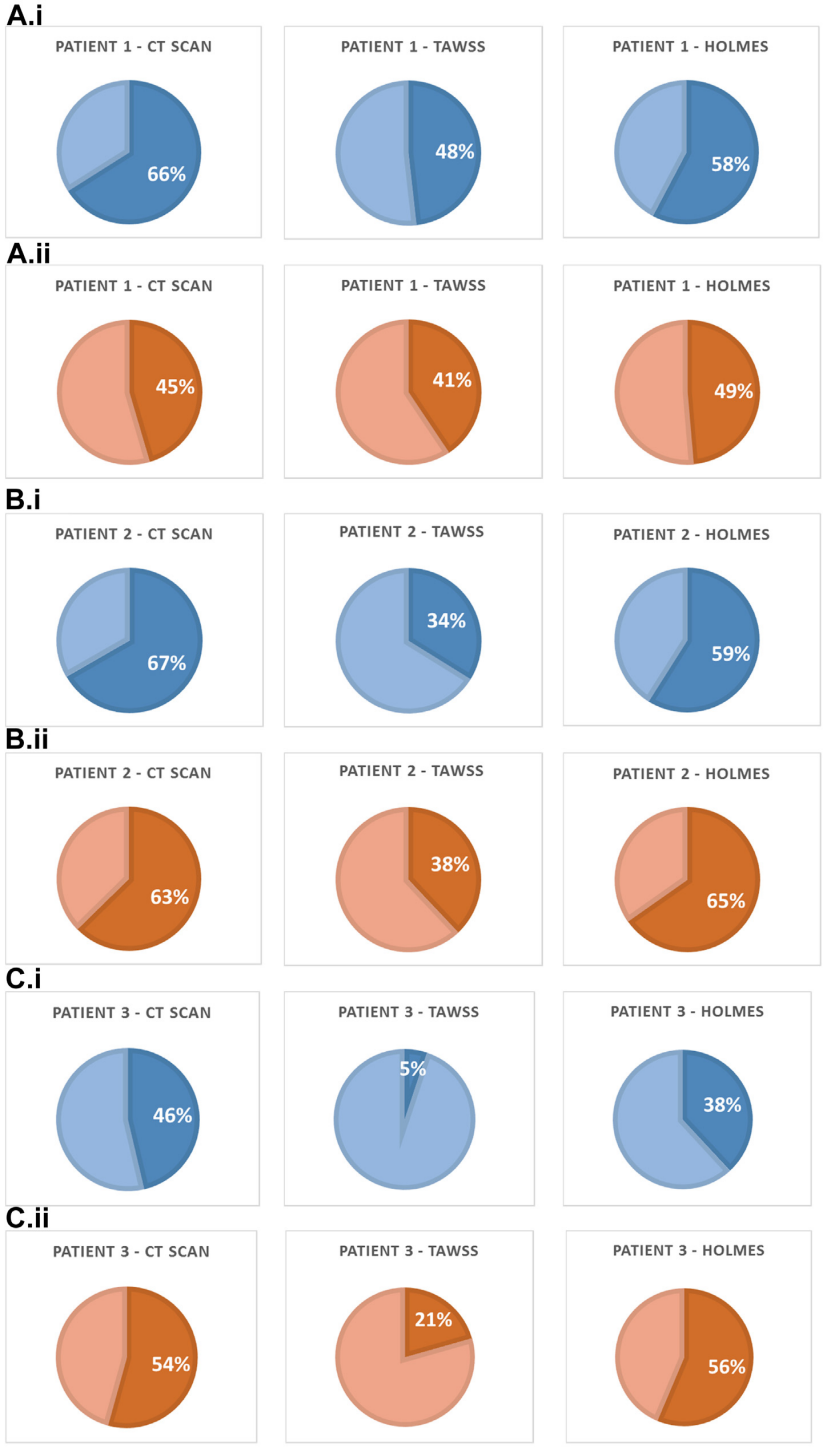

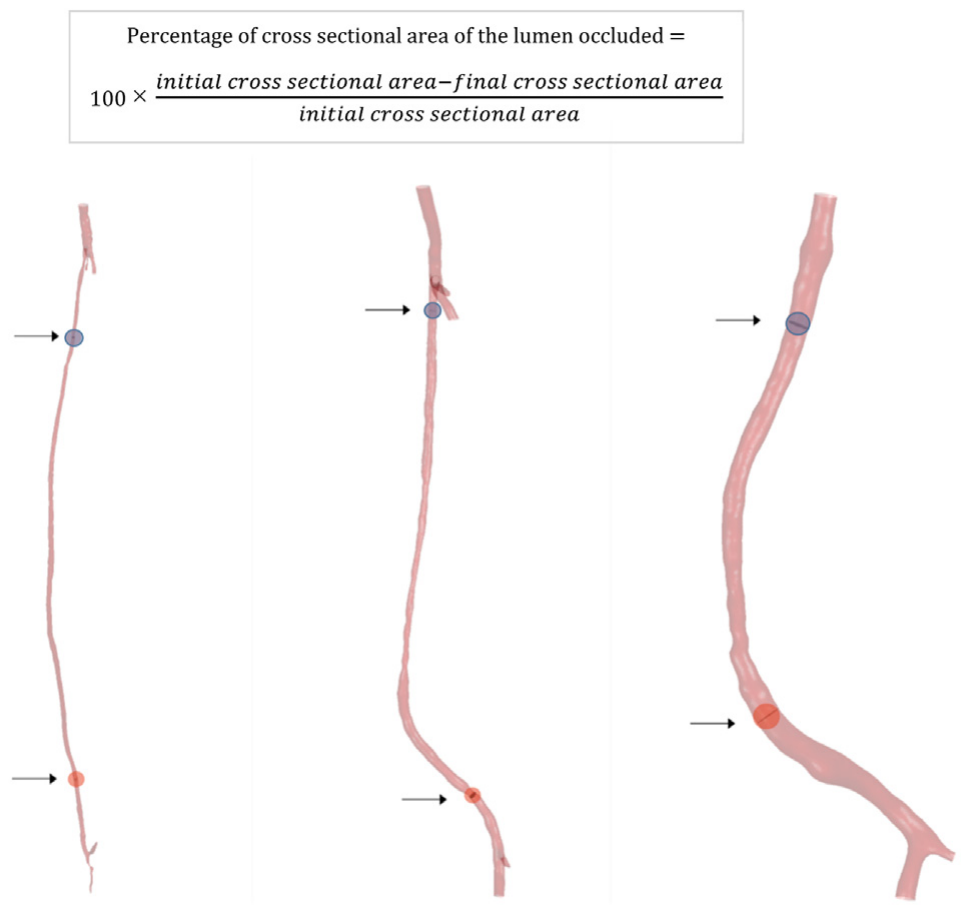
Results of the simulations in percentage cross-sectional area occupied by neointimal hyperplasia (NIH) at the most severely affected locations after the time of NIH development for each patient using the non-newtonian viscosity model and the two different wall shear stress indices (time-averaged wall shear stress [*TAWSS*] and highly oscillatory, low magnitude shear [*HOLMES*]). **A.i,** Patient 1, proximal anastomosis. **A.ii,** Patient 1, distal anastomosis. **B.i,** Patient 2, proximal anastomosis. **B.ii,** Patient 2, distal anastomosis. **C.i,** Patient 3, proximal anastomosis. **C.ii,** Patient 3, distal anastomosis. **D,** Calculations of stenosis at the locations most severely affected by NIH. *CT,* Computed tomography.

Percentage differences between the initial and remodeled lumen areas were calculated and are displayed in Fig 3, with positive and negative predictive values (computed for significant levels of stenosis, >50%, as defined in Shaalan et al^17^) for the model's simulations of stenosis reported in Supplementary Table IV. In all the cases simulated, predicted stenoses were confirmed by the CT scan data, and only one of the bypasses showed a considerable amount of NIH in the midregion of the graft that was not captured by the model because this area coincided with an area of pre-existing vein graft stenosis, another cause of NIH.^18^

**Supplementary Fig 4:**
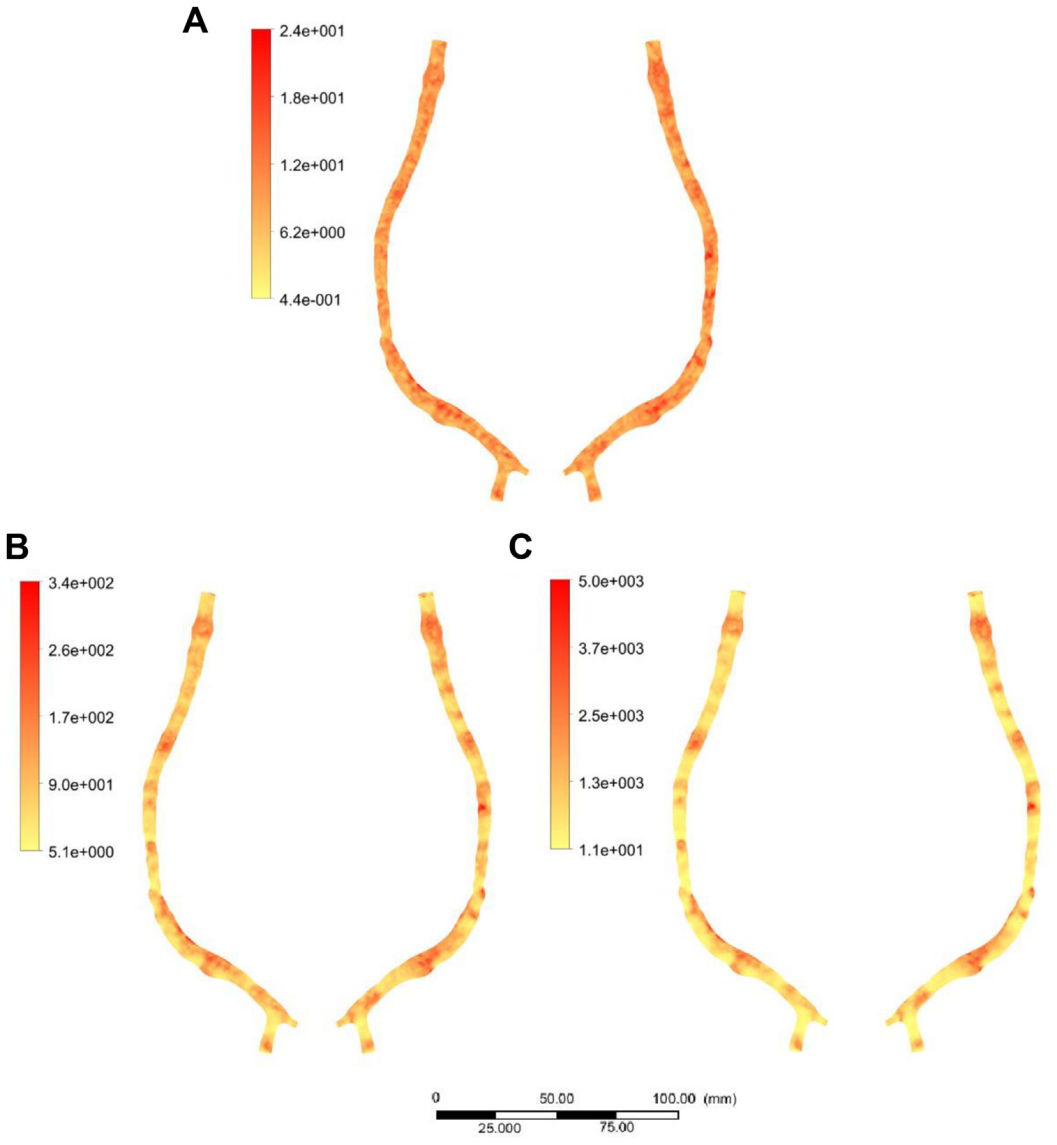
Predicted evolution of smooth muscle cells per baseline value (363 cells/mm^2^) in the intimal layer of the bypass at different time points during the neointimal hyperplasia (NIH) phase (patient 3, non-newtonian case using the highly oscillatory, low magnitude shear [HOLMES] index). **A,** Snapshot at 146 days after intervention. **B,** Snapshot at 438 days after intervention. **C,** Snapshot at 730 days after intervention.

**Supplementary Fig 5:**
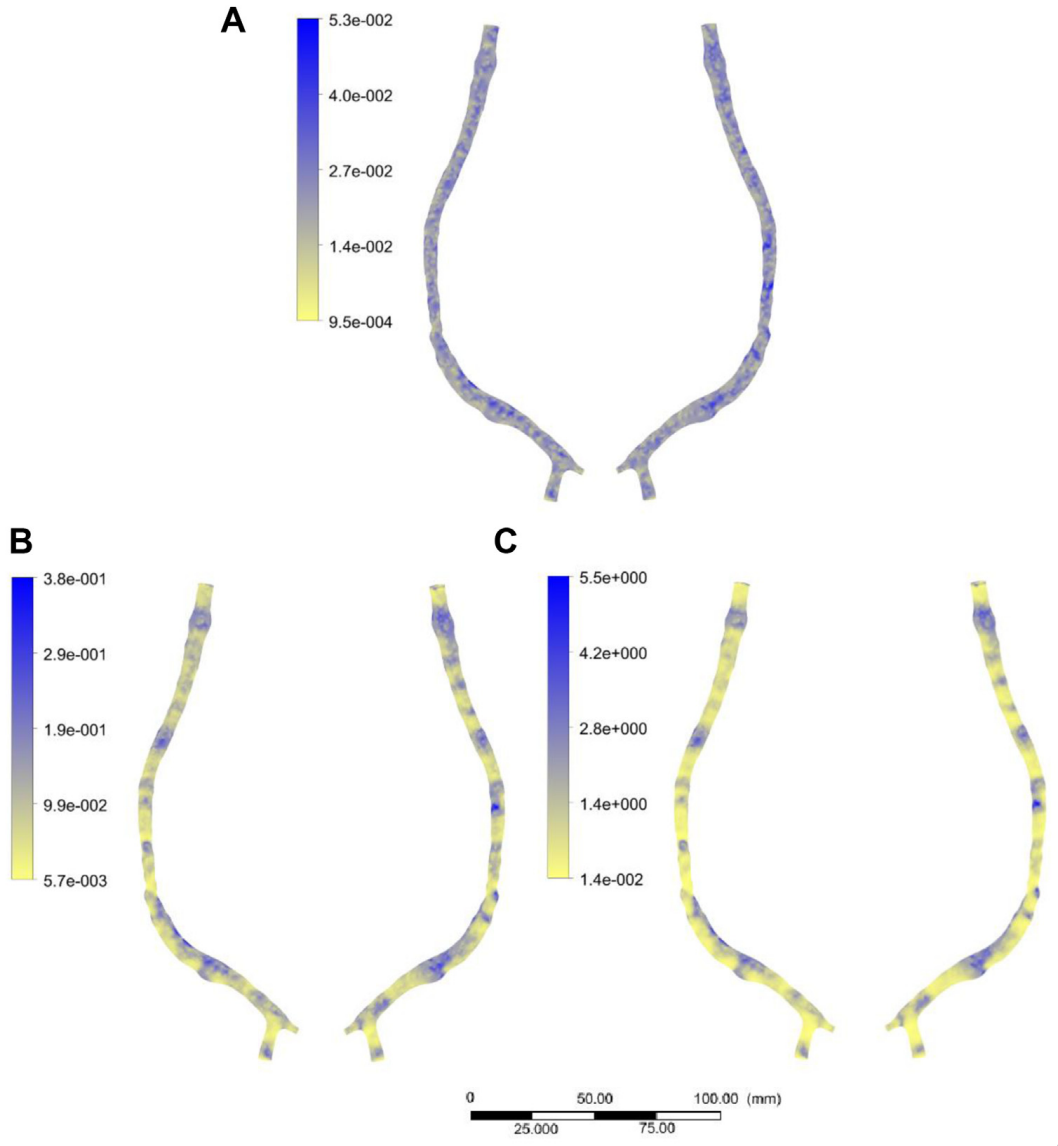
Predicted evolution of collagen per baseline value (1.67 μg/mm^2^) in the intimal layer of the bypass at different time points during the neointimal hyperplasia (NIH) phase (patient 3, non-newtonian case using the highly oscillatory, low magnitude shear [HOLMES] index). **A,** Snapshot at 146 days after intervention. **B,** Snapshot at 438 days after intervention. **C,** Snapshot at 730 days after intervention.

Supplementary Figs 4 and 5 summarize simulation results of the mathematical model used to describe cell proliferation and collagen turnover.^7^ To the authors' best knowledge, values for collagen and smooth muscle cell composition in vivo are not reported in the literature; however, the figures show an increasing number of smooth muscle cells and collagen in areas of low wall shear and high oscillations, which agrees with findings widely reported.^19^

Finally, analysis of graft geometry was performed to measure curvature, torsion, and tortuosity using the Vascular Modeling Toolkit^20^^,^^21^ (www.vmtk.org). Values of torsion and tortuosity were calculated at specific locations of interest and presented the highest differences among NIH-prone locations (up to 99.3%; Supplementary Table V).

## Discussion

**Supplementary Fig 6:**
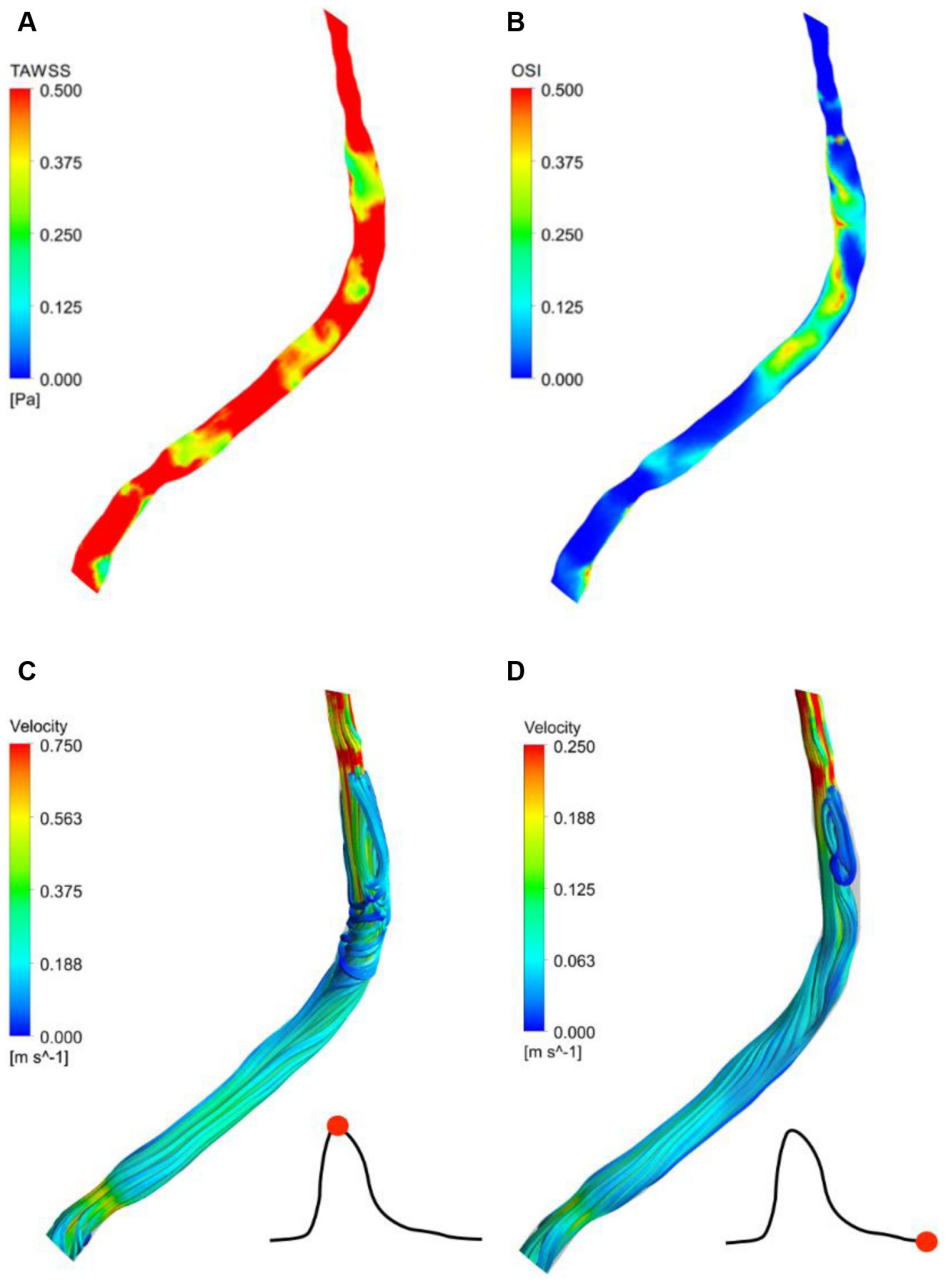
Contour plots of time-averaged wall shear stress (*TAWSS*) and oscillatory shear index (*OSI*) and velocity streamlines at peak systole and end diastole in patient 2. **A,** TAWSS. **B,** OSI. **C,** Velocity streamlines at peak systole. **D,** Velocity streamlines at end diastole.

These results highlight the impact of two different measures of wall shear stress, TAWSS and HOLMES, and the importance of the interaction between TAWSS and OSI in NIH progression (Supplementary Fig 6). In all cases, the simulation model *correctly predicted* areas of NIH growth, with values that were similar to the stenoses observed in the CT scans with use of the HOLMES index, with a maximum discrepancy (presented as percentage area) of 8% between stenosis values observed in patients 1 to 3 compared with CT scans (Fig 3; Supplementary Table IV). With use of TAWSS, not all NIH-stenotic regions are predicted, and for those that are, the amount of luminal narrowing is consistently underestimated and sometimes by a significant amount (as in the case of patient 3) with a reported difference in terms of NIH growth area of 41% (Fig 3; Supplementary Table IV). This suggests that TAWSS is a less reliable metric compared with HOLMES to estimate both plaque location and the degree of stenosis in vein grafts.

CFD has been used to analyze the hemodynamics of grafts for multiple cardiovascular procedures, such as endovascular repair,^22^ carotid endarterectomy,^23^ and arteriovenous fistula.^24^ Much less research has been reported on mechanisms of failure of peripheral bypasses, with most reported work focused on design optimization.^25^ This preliminary study of three patients couples CFD analyses with a model of smooth muscle cells and collagen turnover, including growth factor and nitric oxide production.^7^ The results show a change from an initial, predominantly homogeneous distribution of smooth muscle cells and collagen at day 146 to a localized area of growth corresponding to areas in which the graft experienced low shear and oscillations (Supplementary Figs 4 and 5). In addition, our analysis shows that morphometric indicators alone might not be enough to identify successful grafts. For instance, torsion has been observed to be one of the key factors affecting large-vessel hemodynamics,^26^ and our results agree with these findings. However, the results present too high variability and thus are difficult to generalize.

This novel analysis of vascular remodeling is significantly more effective in localizing areas of NIH progression compared with use of only hemodynamic or morphometric indices. By incorporating the effects of hemodynamic variables on the biologic behavior of smooth muscle cells and collagen as well as performing hemodynamic analysis on patient-specific geometries, the model enables the calculation of stenosis due to NIH progression for each individual patient. We believe that such models represent a valid alternative to experimental tests in vitro and in vivo to assess basic hypotheses on the development of NIH. This framework can currently be used to simulate different flow conditions and graft geometries and suggests improvements based on the analysis. With further refinement and validation on larger cohorts of patients, it has the potential to be developed into a tool for surgical planning.

### Limitations

Multiple factors affect NIH remodeling, such as pre-existing valve lysis, as demonstrated by the results for patient 2, which should be included in future simulation frameworks. The framework could also be improved by considering additional biologic mechanisms related to growth factors, extracellular matrix components, and endothelial cells, all of which have been shown to influence NIH.^1^ Patient-specific biomarkers such as C-reactive protein, inflammatory cytokines, and adhesion molecules^27^^,^^28^ also correlate with severity of NIH. Patient-specific characteristics, such as smoking and medication use, as well as other clinical conditions, such as hypertension, have been assumed not to affect the patients in the current analysis; however, they might influence the response of individual patients and should be considered in future developments of the model. The voxel size in the CT scans is close to the order of magnitude of the geometries considered, and this might have an impact on the results. Another limitation with regard to the clinical images was the availability of CT scans at the final time point only, which resulted in the need to adopt the virtual removal technique, as described in the Methods; a prospective study is needed for further validation. Finally, the model might also be limited by the assumption of rigid arterial walls, and the impact of this assumption needs to be verified through further analyses.

## Conclusions

In the application of a multiscale model of NIH on three different patient-specific cases, the HOLMES index was the best predictor for the location of NIH progression that corresponded to developing stenoses identifiable in CT scans compared with TAWSS. Use of the HOLMES index with a non-newtonian model of viscosity in the CFD analysis combined with the biochemical model of NIH allowed better prediction of which locations would develop NIH. The amount of NIH growth was close to reported CT values (Supplementary Table IV). Our analysis also demonstrated how multiscale modeling may play an important role in the postrevascularization management of peripheral artery disease patients and specifically in delineating those at risk for development of NIH.

Our findings of this retrospective study need prospective validation with a robust study design to confirm a generalization of these results.

## Figures and Tables

**Supplementary Table I:** A summary of shear stress indices used in this study

Index	Formula	Meaning and relevance in cardiovascular disease
TAWSS	$\frac{1}{T}\int_{0}^{T}\left|{\vec{\tau }}_{w}\right|dt$ where *T* is time and τω is wall shear stress	A measure of wall shear stress for one cardiac cycle; values <0.5 Pa co-localize with areas of neointimal thickening
OSI	$0.5\left(1-\frac{\left|\int_{0}^{T}{\vec{\tau }}_{w}dt\right|}{\int_{0}^{T}\left|{\vec{\tau }}_{w}\right|dt}\right)$	Used to identify areas where the flow deviates most from its average direction, that is, where it oscillates most
HOLMES	TAWSS (0.5 - OSI)	A modified TAWSS, highlighting regions of low shear and modulated by high oscillations

*HOLMES,* Highly oscillatory, low magnitude shear; *OSI,* oscillatory shear index; *TAWSS,* time-averaged wall shear stress.

**Supplementary Table II:** Summary of the parameters used for the two-element Windkessel models

	R_1_	R_2_	R_3_	R_4_	C_1_	C_2_	C_3_	C_4_
Patient 1	12.5	9.2 × 10^2^	1.5 × 10^3^	1.3 × 10^3^	2 × 10^−2^	1.5 × 10^−2^	1.5 × 10^−2^	1.5 × 10^−2^
Patient 2	25	2 × 10^2^			2.78 × 10^−2^	3.02 × 10^−2^		
Patient 3	13.9	1.89 × 10^2^			2 × 10^−2^	1.72 × 10^−2^		

R units, mm Hg s mL^−1^; C units, mL mm Hg^−1^.

**Supplementary Table III:** Overview of the model characteristics

	Patient 1	Patient 2	Patient 3
CFD model			
Mesh elements	1.6M	380k	200k
Mesh sensitivity analysis			
Coarse mesh	700k	120k	70k
Medium mesh	1.6M	380k	200k
Refined mesh	2.6M	600k	500k
Difference in velocity at outlets between refined and medium mesh (<2% required for mesh convergence)	1.6%	0.9%	1.9%
Assumptions	Non-newtonian model of viscosity (Carreau-Yasuda) Flat profile at the inlet Two-element Windkessel models at the boundary conditions		
NIH progression model			
Input	TAWSS or HOLMES		
Output	NIH volume $V_{i}=\left(S_{i}+Q_{i}\right)\times \rho_{s}^{-1}+C_{i}\times \rho_{c}^{-1}$ where *i* is a suffix referring to the intimal layer; *S*, smooth muscle cells; *Q*, quiescent cells; *C*, collagen; ρs, cell density (cells/m^3^); and ρc, collagen density (cells/m^3^).		

*CFD,* Computational fluid dynamics; *HOLMES,* highly oscillatory, low magnitude shear; *NIH,* neointimal hyperplasia; *TAWSS,* time-averaged wall shear stress.

**Supplementary Table IV:** Results from the computed tomography (*CT*) scans and simulations for the three patients, with percentage stenosis and identification of significant stenosis using a threshold of >50%

	Stenosis, %						
	CT scans	TAWSS			HOLMES		
		Plug inflow	Parabolic inflow	Non-newtonian viscosity	Plug inflow	Parabolic inflow	Non-newtonian viscosity
Patient 1							
Proximal	66.0	49.8	49.8	48.2	58.4	58.4	57.7
Distal	48.9	43.5	43.5	42.8	50.3	50.3	50.0
Patient 2							
Proximal	66.7	44.2	44.3	33.9	66.1	66.2	58.9
Distal	62.7	47.8	47.8	38.0	69.0	69.0	65.3
Patient 3							
Proximal	46.3	11.3	7.3	5.1	39.2	39.9	38.2
Distal	54.3	25.2	27.0	20.6	56.1	54.7	56.3

Did >50% stenosis develop?							
	CT scans	TAWSS			HOLMES		
		Plug inflow	Parabolic inflow	Non-newtonian viscosity	Plug inflow	Parabolic inflow	Non-newtonian viscosity
Patient 1							
Proximal	✓	⨯	⨯	⨯	✓	✓	✓
Distal	⨯	⨯	⨯	⨯	✓	✓	⨯
Patient 2							
Proximal	✓	⨯	⨯	⨯	✓	✓	✓
Distal	✓	⨯	⨯	⨯	✓	✓	✓
Patient 3							
Proximal	⨯^a^	⨯^a^	⨯^a^	⨯^a^	⨯^a^	⨯^a^	⨯^a^
Distal	✓	⨯	⨯	⨯	✓	✓	✓
		PPV: 0%	PPV: 0%	PPV: 0%	PPV: 4÷5 = 80%	PPV: 4÷5 = 80%	PPV: 4÷4 = 100%
		NPV: 2÷6 = 33%	NPV: 2÷6 = 33%	NPV: 2÷6 = 33%	NPV: 1÷1 = 100%	NPV: 1÷1 = 100%	NPV: 2÷2 = 100%

*HOLMES,* Highly oscillatory, low magnitude shear; *NPV,* negative predictive value; *PPV,* positive predictive value; *TAWSS,* time-averaged wall shear stress.

Use of TAWSS alone was not sufficient to identify areas of >50% stenosis, whereas all areas were identified with the use of HOLMES combined with the non-newtonian model of viscosity while also reducing the false-positive value.

a In patient 3 at the proximal anastomosis, stenosis measured in the CT scans was 46.3%; as a result, no severe stenosis was identified.

**Supplementary Table V:** Morphometric analysis of the three grafts

	Curvature, mm^−1^	Torsion, mm^−1^	Tortuosity
Patient 1 proximal	0.034589	0.001381	0.011302
Patient 1 distal	0.026341	0.11598	0.012554
Patient 2 proximal	0.027653	0.101641	0.011055
Patient 2 distal	0.029005	0.0444	0.121906
Patient 3 proximal	0.034212	0.008087	0.012636
Patient 3 distal	0.037476	0.000842	0.051892
